# National Prescribing Patterns in Stoma Care Across the United Kingdom: A Prescription Cost Analysis with Health Policy Implications

**DOI:** 10.3390/healthcare14183019

**Published:** 2026-09-15

**Authors:** Victoria Gilpin, Cliodhna McCann, Niamh Magee, Erika J. Rosbotham, Sebastian Leonties, L. Kirsty Pourshahidi, Chris I. R. Gill, Ellen E. A. Simpson, Karl McCreadie, Sarah-Jane Nelson, James Davis

**Affiliations:** 1 School of Engineering, Biomedical Engineering, Ulster University, Belfast BT15 1ED, Northern Ireland, UK; gilpin-v@ulster.ac.uk (V.G.); mccann-c94@ulster.ac.uk (C.M.);; 2 School of Biomedical Sciences, Ulster University, Coleraine BT52 1SA, Northern Ireland, UK; magee-n20@ulster.ac.uk (N.M.); e.rosbotham@ulster.ac.uk (E.J.R.); k.pourshahidi@ulster.ac.uk (L.K.P.); c.gill@ulster.ac.uk (C.I.R.G.); 3 School of Psychology, Ulster University, Coleraine BT52 1SA, Northern Ireland, UK; eea.simpson@ulster.ac.uk; 4 School of Computing, Engineering and Intelligent Systems, Ulster University, Derry/Londonderry BT48 7JL, Northern Ireland, UK; k.mccreadie@ulster.ac.uk; 5 Regional Intestinal Failure Service, Belfast Health and Social Care Trust, Belfast BT12 6BA, Northern Ireland, UK; sarahjane.nelson@belfasttrust.hscni.net

**Keywords:** stoma, ostomy, prescribing, health policy, prescription cost analysis, National Health Service

## Abstract

**Objectives:** The prevalence of stoma formation in the United Kingdom has risen markedly over the past decade, yet comparatively little is known about national prescribing patterns for ostomy products. A retrospective cross-sectional observational study was conducted in which the UK Prescription Cost Analysis (PCA) data for 2023 were examined to assess what information can be extracted from these datasets, to evaluate inter-nation variation in prescribing, and identify areas warranting further health economics and policy investigation. **Methods:** PCA data for calendar year 2023 were collated from the four devolved UK health services. Data on product type, manufacturer, quantities dispensed, and cost were compared across nations and product categories. **Results:** Total UK prescription spend on ostomy products in 2023 was £457 million, 3.4% of overall NHS prescription expenditure. Pouches accounted for 72% (£329 million) of spend, with the remainder supporting peristomal skin health and appliance adhesion. Estimated average per-patient spend was modelled and varied substantially by nation, from £1073 in Wales to £2354 in England, against a UK average of £2243. Persistent prescribing of ancillary items no longer recommended for routine use (filters, deodorants, bag covers) accounted for an estimated £ 8 million (>2% of spend). **Conclusions:** PCA data represent an underutilized resource for population-level analysis of ostomy care. Marked inter-nation variation in expenditure, uncertainty surrounding ostomate population estimates, and the continued prescribing of ancillary items not recommended for routine use identify important evidence gaps and generate hypotheses for future research with direct relevance to prescribing policy, resource allocation, and equitable access to care across the United Kingdom.

## 1. Introduction

**Figure 1:**
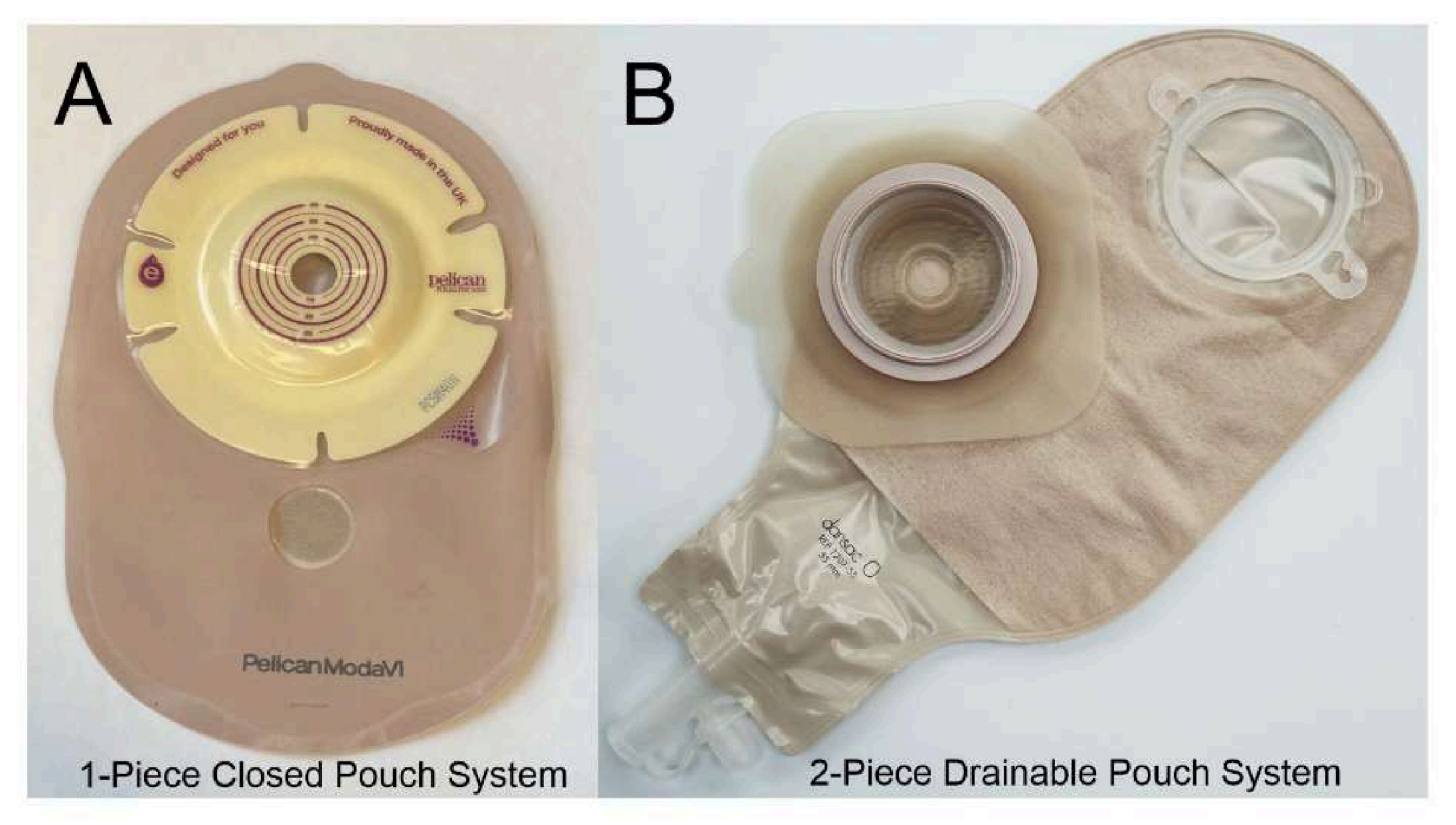
Comparison of a 1-piece closed pouch system (**A**), in which the adhesive flange is directly attached to the pouch, and a 2-piece drainable system (**B**), where the pouch and flange are physically separate.

Surgical diversion of the bowel or ureters to create an opening in the abdomen (stoma) through which bodily waste can be eliminated is a common procedure in the treatment of various gastrointestinal and bladder conditions. Irrespective of whether the stoma is a colostomy, ileostomy or urostomy, the waste is almost invariably collected in a disposable pouch. The prevalence of stoma formation in the United Kingdom has increased markedly in recent decades. In 2010, an estimated 100,000 individuals were living with a stoma; by 2023, this figure has been estimated to be between 176,000 and 205,000 [1,2,3,4]. This is largely attributable to the rising incidence of inflammatory bowel disease (IBD) and colorectal cancer (CRC), with King et al. (2020) reporting a sustained annual increase in IBD diagnoses of 2–3% over the preceding two decades [5]. As the ostomate population expands, it has become increasingly clear from a policy perspective that the clinical and economic burden of stoma management requires careful scrutiny. Central to ostomy care is the stoma pouch, whose management is associated with complications that can substantially impair patient quality of life (QoL) [6,7,8,9]. Optimal outcomes require consideration of factors extending well beyond the initial surgical procedure, encompassing physiological, microbiological, biochemical, and nutritional variables, alongside the pouch’s material properties and design [10]. Considerable inter-individual variability in stomal morphology and patient need renders a universal pouch solution impractical and, as a consequence, the ostomy market supports an extensive product portfolio [10]. The two core design approaches commonly used in ostomy management are based on either a 1-piece or 2-piece pouch format, as illustrated in Figure 1. These can be further categorized as closed or drainable systems. In general, individuals with a colostomy typically use closed systems, where the output is more solid and stool-like. By contrast, output from an ileostomy tends to be more frequent and more liquid, so a drainable system is typically used. In the case of a urostomy, the stoma is usually formed from a section of the small intestine; although some mucus may be present, the output is essentially urine, making a drainable bag a necessity.

Despite the breadth of available products, pouch-related complications such as leakage, peristomal skin damage, flatus management difficulties, and pancaking remain prevalent [6,8]. A range of ancillary products has been developed to address these issues, and the global ostomy market is now valued at $3.9 billion and is projected to reach $5.3 billion by 2030 (CAGR: 5.5%) [11]; yet, comparatively little is known about prescribing trends across product categories within national healthcare systems.

In the UK, stoma care products are supplied almost exclusively via prescription, and national prescribing databases therefore represent a valuable resource for population-level analysis. Prescription Cost Analysis (PCA) data, freely available from each devolved nation’s health service [12,13,14,15], provide granular information on product type, manufacturer, quantities dispensed, and costs, potentially revealing trends in product utilization, patient preference, and evolving care pathways—a complementary, and potentially more objective, perspective than the QoL studies and practitioner surveys that typically inform changes in ostomy care. This study reports a systematic examination of UK PCA data for 2023, assessing what information can be extracted from these datasets, evaluating inter-nation variation in prescribing patterns, and identifying areas warranting further health economics and policy investigation.

## 2. Methods

### 2.1. Data Sources

Prescription data are compiled separately within each of the four devolved nations of the UK: the NHS Business Services Authority (NHSBSA) for England [12], Public Health Scotland (PHS) [13], the NHS Wales Shared Services Partnership (WSSP) [14], and Northern Ireland’s Health and Social Care Business Services Organization (HSCBSO) [15]. Despite differences in how each authority manages and presents data, there is sufficient commonality and granularity for collation and cross-national comparison. Data are generally compiled by calendar and financial year, or as monthly statistics in WSSP’s case [14], detailing product type, manufacturer, quantities dispensed, and costs. NHS Wales reports monthly prescription data, in contrast to the annual statistics presented by England, Scotland, and Northern Ireland. In all four cases, however, the summary data are listed under common headings defined by the British National Formulary (BNF). These are represented as BNF chapters and subsections. As such, stoma appliances appear throughout as Chapter 23, with distinct subsections. For example, “Adhesive Discs/Rings/Pads/Plasters” is recorded as Section 5, whereas products listed under “Bag Covers” are denoted as Section 25. These headings are consistent across the PCA data for all four nations and provide direct comparability at section-heading level. Product-level data, however, required manual inspection of individual items, with prescription descriptions enabling the collation of group totals. Product names are commonly used in the descriptive labels of each prescription line and were used to derive manufacturer-level summary figures.

### 2.2. Scope and Limitations of the Dataset

No formal ethical approval was required, as the datasets are publicly available and fully anonymized. The data used here relate to 2023, the most current and complete dataset covering the whole UK at the time of writing, enabling direct comparison between the four nations. PCA data does not provide any indication of unique prescription request data and only reveals the total number of items prescribed. As such, it is not possible to discern how many individuals have requested a given product and therefore there is no scope for the direct determination of the stoma population from the PCA data itself. The prescription data therefore offer snapshots of products supplied across the UK.

An important limitation is that the datasets relate only to items dispensed in the community: prescriptions written in hospitals but dispensed in the community are included, but those supplied directly by hospital pharmacies, issued and dispensed within prisons, or obtained via private prescription are excluded. PCA data captures only community prescribing. Although direct-to-patient (DTP) and industry-sponsored dispensing appliance contractors (DAC) supply individuals, these generally fall within the community reimbursement framework. Some DTP contracts, however, bypass community prescribing reimbursement. Health board-level DTP procurement is used in secondary care and typically would include situations where ostomates were awaiting transfer to primary/community care and after specialist consultations where products would be prescribed, again prior to transfer. However, it should be noted that most non-PCA DTP contracts relate to medicines or biologics rather than appliances.

## 3. Results

### 3.1. National Overview

The total UK prescription spend on ostomy products in 2023 amounted to £457 million, 3.4% of overall UK NHS prescription expenditure. It must be noted that within the devolved nations of the UK, there are no registries from which the stoma population (or individual stoma type) can be directly estimated. Recent estimates of the UK stoma population vary widely from 176,824 to 205,000 [1,2,3,4]. The East England NHS Collaborative Procurement Hub StoMap Programme Baseline Report (2019) estimated the total ostomate population in the UK at 176,824 (1 in 500) and was based on a combination of Collaborative Procurement Partnership and British Healthcare Trade Association (BHTA) data [1]. An ostomate population of 205,000 offered by Hodges (2022) is the most recent and often quoted estimate, but it is also derived from BHTA figures, which would equate to 1 in every 335 people [2,3,4]. Both figures are regularly repeated in the literature to highlight the stoma prevalence within the UK, but as both originate from commercial data rather than clinical registries, transparency in their estimation is lacking. The estimates are, however, similar to the European and US figures (Table 1), albeit with the caveat that each uses different reporting methodologies and, like the UK figures, is often similarly opaque.

Using the StoMap and Hodges (2022) figures as lower and upper bounds [1,3], respectively, Table 2 presents a breakdown of average prescription costs per ostomate based on the 2023 PCA data. The Scottish Stoma Forum (2023) estimated 19,993 ostomates in Scotland, slightly higher than the figure derived here, though their estimated average annual per-patient spend of £1787 is comparable to the £1726 reported herein [20]. Geographical variation in ostomate prevalence inevitably exists beyond the 1 in 335 statistic and reinforces the fact that the average per-patient cost calculations are an estimate. It is also important to recognize that the total stoma population comprises several subgroups, typically colostomy, ileostomy, and urostomy, each of which uses different products. As with total population size, no formal databases are available to determine the relative proportion of ostomates within each subgroup. Mthombeni et al. (2023) conducted a retrospective Clinical Practice Research Datalink (CPRD) study linked to Hospital Episode Statistics (2009–2018) and, in a sample of 9367 people in England, estimated the colostomy/ileostomy/urostomy ratio as 65/26/9, respectively [21]. Burch (2014) estimated a prevalence ratio of 53–45/38/10–18 and highlights the considerable variability that can arise in such studies [22]. Given the additional uncertainty associated with off-label use of stoma products, the estimated average spend per patient should be interpreted with considerable caution. This is a distinction that is critical given that NHSBSA data are intended to help inform policy [12].

The StoMap Baseline Report estimated mean annual prescription expenditure at £2078 per ostomate [1]; given the four-year interval between that report and the present analysis, this figure is broadly consistent with the estimates derived here. However, these values should be interpreted as population-level averages rather than as representative of individual prescribing costs. In practice, annual prescription expenditure is likely to be substantially lower for many ostomates, particularly those who experience few complications and have stable appliance requirements. Accordingly, the calculated average cost per ostomate is necessarily a derived measure and should not be assumed to reflect routine individual expenditure. The National Stoma Quality Improvement Group (NSQIG) working group report indicated that annual prescription costs typically range from £780 to £2300 per patient, although, as discussed later, costs may increase considerably in complex cases, exceeding £6000 per annum [23].

The bulk of UK prescription costs accrue from England, reflecting its substantially greater ostomate population (85%) (Table 2). The average annual spend in Wales appears anomalous, at less than half of the UK average per ostomate (48%). Given the uncertainty surrounding the size and distribution of the stoma population across the UK, this estimate should be interpreted with considerable caution. Nevertheless, it warrants closer scrutiny. If Welsh per-patient spend matched the UK average, the implied prevalence would be approximately 1 in 700 rather than 1 in 335, raising questions about regional variation in stoma prevalence, prescribing guidance, clinical practice, or service configuration.

**Figure 2:**
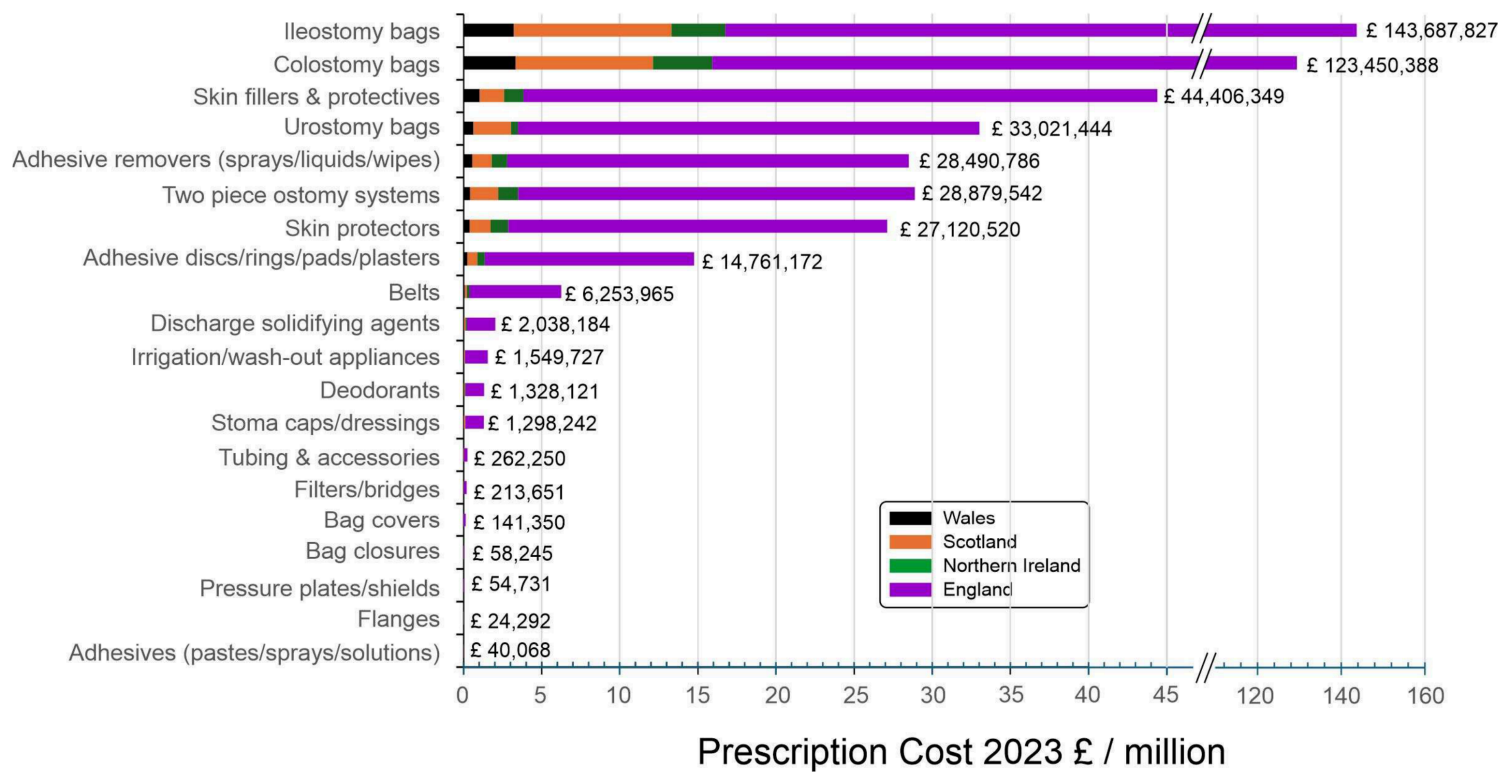
Prescription Cost Analysis (PCA) summary data for the UK National Health Service in 2023. (Adapted from the collated UK PCA 2023 data).

Figure 2 details the annual spend across the stoma product portfolio. Pouch supply dominates expenditure (£329 million, 72%), but a closer analysis revealed inter-nation differences in pouch type: in Wales, 1-piece ileostomy and colostomy pouches accounted for 32% and 33% of annual spend respectively, versus UK averages of 31% and 27%. Since ileostomy management is generally costlier than colostomy management given greater output frequency and volume [16,24], a relative reduction in ileostomy prevalence in Wales would be expected to lower both pouch costs and ancillary spending.

Guidance over the supply of ostomy accessories is managed through local integrated care boards (ICBs) or regional health trusts, and their aim is to standardize practice rather than provide blanket restrictions on particular products. Nevertheless, some products are typically categorized as being “***should not be routinely prescribed***” or “***only to be prescribed if requested by a specialist stoma nurse***” [25]. Typical examples are highlighted in Table 3.

The prescribing of stoma filters and bridges is generally recommended only following consultation with a specialist stoma care nurse (SCN), where a clear clinical benefit to the ostomate can be established [26,27,28]. Consistent application of the “not for routine prescription” guidance outlined in Table 3 across the UK in 2023 may therefore have generated estimated savings of approximately £8 million (>2%). Extending this restricted category to include products classified as “Adhesive removers” would have increased the estimated savings to just over £36 million. However, although the removal of such items is frequently proposed as a cost-containment measure, the overall net financial impact is relatively modest, and any restrictions should be implemented cautiously, with SCN oversight, to ensure that products providing genuine clinical value to individual patients are not withdrawn. The Ostomy Life QoL survey of 4000 ostomates (2015) identified leakage as a primary concern (91%), with comparable findings in the 2019 follow-up [7,29]. Osborne et al. (2022), surveying 301 respondents, found leakage (90%) and peristomal skin complications (PSCs) (82%) remain the most significant sources of anxiety for ostomates [8], suggesting that despite advances in pouch design [8], fundamental clinical challenges remain unresolved.

### 3.2. Stoma Pouch Supply in England

**Figure 3:**
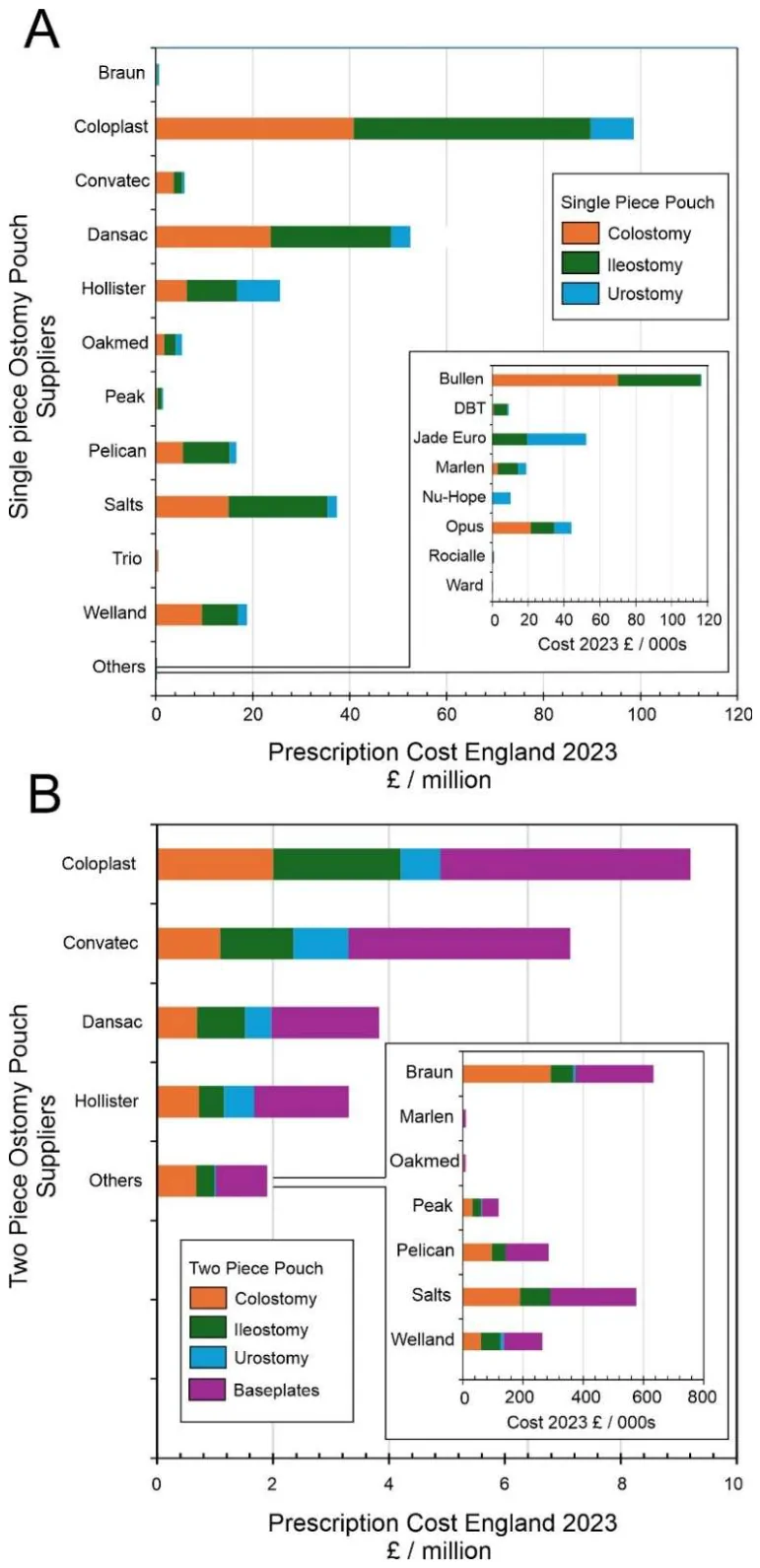
Summary of the 2023 prescription spend on (**A**) single-piece pouches and (**B**) two-piece pouch systems for England. (Based on data extracted from the NHSBSA Prescription Cost Analysis).

A more detailed examination of pouch supply was undertaken using prescription data for England and a breakdown by manufacturer and by type is shown in Figure 3. England was selected on the basis that it accounts for the majority of PCA expenditure and, given its larger stoma population, was expected to provide coverage of the principal manufacturers of stoma pouches and associated accessories. Pouch costs for England alone in 2023 amounted to £289 million, 71% of its annual prescription spend (£405 million). It must be reiterated that there is no universal pouch type; selection depends on physical factors (stoma size, shape, output consistency and volume, peristomal reactions to adhesive baseplates) and personal factors of esthetics and practicality (profile, color, materials) [10]. Typical prescribing guidelines for each stoma type are summarized in Table 4.

While both 1-piece and 2-piece options are available, the 1-piece pouch is most commonly prescribed (£264 million, 65%). For colostomy patients, closed 1-piece pouches are recommended by the UK’s National Institute for Health Care Excellence (NICE) as the most cost-effective standard unless complications justify alternatives [30]. These systems typically offer greater simplicity, particularly for patients with limited dexterity and also result in a lower body profile due to the absence of a coupling mechanism. The relative spend on different pouch types in England is shown in Figure 3; the 2023 data correspond to over 67 million 1-piece and 6.5 million 2-piece pouches supplied.

The supply of 1-piece systems is broadly split between colostomy and ileostomy types (31.9 million vs. 30.5 million), though ileostomy pouches (£127 million, 31%) cost more overall than colostomy pouches (£108 million, 26%). Caution is, however, warranted, as a pouch typically designated for one stoma type may be used for another—for example, high-output ileostomates (>1.5 L/24 h) often use the higher capacity drainable urostomy pouches given their output’s higher water content [31]. In general, 1-piece pouches are less expensive (£1.18–£4.00 each) than the corresponding 2-piece systems, whose additional components can be expected to raise unit cost. However, the longer-term cost-effectiveness of 2-piece systems merits consideration in certain scenarios given their potential to reduce skin stripping, peristomal irritation, and associated PSC risk.

As an illustration, a Dansac Nova Life 1-piece colostomy pouch (45 mm) was reimbursed at £3.12 in 2023, versus £4.89 for its 2-piece equivalent (pouch: £1.45; flange: £3.44). Following the guidelines in Table 4 (3x daily pouch change; 3-day flange wear), this implies a monthly cost of £280 for the 1-piece system versus £165 for the 2-piece system. The 2-piece saving, however, depends on the flange remaining in situ for several days; it diminishes sharply if flanges are changed more frequently due to adhesion, irritation or leakage issues. For ileostomy pouches, however, the benefits of a 2-piece system are less clear. In these cases, a drainable pouch would typically be used for up to three days whether supplied as a 1- or 2-piece system; therefore, in the absence of other clinical considerations, the 1-piece option is likely to be more cost-effective. A possible confounding factor is the integrated pouch filter. Filter blockage is frequently reported in QoL studies, and when more frequent pouch replacement is required, a 2-piece system may offer advantages. While there is anecdotal evidence of filter issues, there remains little quantifiable evidence on the prevalence of the problem or on the comparative effectiveness of 1- and 2-piece systems.

### 3.3. Peristomal Skin Health

Beyond pouches, much of the remaining prescription spend in England in 2023 (£116 million, 25%) was targeted at supporting pouch application and appliance function. Since stomal morphology and skin condition are seldom ideal, there is a diverse range of accessories designed to improve adhesion, fill skin contour irregularities, and protect peristomal skin. Adhesion can be compromised by inaccurate aperture cutting or anatomical features such as scars, creases, and folds [8,32,33,34]. Ideally, the stoma should protrude 1–3 cm above the skin so output drains directly into the pouch [32,35,36], but post-surgical retraction can leave it flush or recessed, increasing the opportunities for the output to track beneath the baseplate [32,35] and degrading the hydrocolloid adhesive [37]. Adhesion failure typically increases pouch consumption, necessitating the use of costlier convex baseplates and can precipitate peristomal skin complications (PSCs) [6,24,38,39,40]. The latter typically arises from irritation, inflammation, and moisture-associated skin damage (MASD) from effluent exposure [37,41]. These can range from mild to debilitating, with reported incidences of 36–88%, particularly in the immediate post-operative period [6,39,40], though they can develop even in well-managed stomas. Ileostomates (25–43%) are more susceptible to PSCs than colostomates (7–20%) [38], since ileal output’s higher liquid and enzyme content penetrates beneath the baseplate more readily. Unfortunately, sustained PSCs can compromise adhesion, exacerbating skin damage and are attributed to increased SCN consultations [8,33,42]. The true burden, however, is likely to be under-reported due to patient stoicism. Nybæk et al. (2009), studying 338 ostomates, found 45% presented with an underlying skin condition, yet only 16% had sought help [43]. Left unresolved, PSCs frequently require clinical intervention, with documented increases in economic burden and hospital readmission, particularly early in recovery; Rolls et al. (2022) estimated additional prescription costs from £258 for mild PSCs to £505 for severe cases [40].

Pre-surgical stoma siting is a key determinant of QoL, reducing leakage and PSC incidence and consequent prescription burden. Cruz et al. (2021) and Pearson et al. (2020) found emergency surgery patients were less likely to receive consultative site evaluation and more likely to develop leakage and PSCs [44,45]. The consequences typically surface post-discharge as the ostomate resumes normal activity and previously undetected skin creases compromise the seal, driving demand for protective products, barrier creams and films, adhesive extenders, and additional pouches [24,39,40].

### 3.4. Inappropriate Prescriptions and Stockpiling

Patient overstocking is frequently cited as a contributor to excess prescription expenditure, though prevention presents complex clinical, logistical, and ethical challenges requiring careful policy consideration [20,23,46,47]. General prescribing guidelines exist for pouch supply [25,26,27,28], and sustained increases in consumption beyond expected levels may indicate appliance unsuitability. A standard prescription typically covers a one-month supply, but inventory can deplete faster due to dietary changes, intercurrent illness, or fluctuations in output, all of which can compromise the seal and accelerate consumption [48]. Even previously stable ostomates may see increased demand from adhesion difficulties, actual or anticipated, or elevated throughput [7,8,33,49]. Since pouches are not available over the counter, ostomates cannot readily supplement supply between prescriptions, so more restrictive policies carry real risk: patients with peristomal irritation may continue wearing a failing appliance to preserve inventory, exacerbating complications and hospitalization risk [6,39,40,50]. Any strategy to reduce excess prescribing must therefore be carefully calibrated.

Insufficient or infrequent clinical review is a recognized contributor to overstocking and inappropriate prescriptions [51]. An audit by Northampton Clinical Commissioning Group found 21% of prescribed items were accessories not meaningfully contributing to improved outcomes. NSQIG (2019) reported a case where targeted review halved one patient’s £5000 annual prescription expenditure. NHS Fife reported savings of £60,000 from structured review of 50 high-expenditure outliers [23], though such cases should not be used to infer systemic inefficiency across the broader population; a proportionate, individualized approach is essential [52]. Approximately 80% of ostomates obtain supplies through a dispensing appliance contractor (DAC) and repeat DAC prescriptions may generate surplus where clinical circumstances change (i.e., stoma reversal) without notification [23]. Although robust national reversal data are lacking, 2017/18 English hospital admission data indicate 32% of stoma surgeries were initially planned as temporary [53], a substantial cohort for whom automated repeat prescriptions may continue beyond reversal.

## 4. Policy Implications

### 4.1. Evolving Prescribing Practices and Waste Reduction Strategies

In most cases, GPs retain primary responsibility for stoma prescriptions. However, the ostomy product portfolio’s breadth and complexity make it unrealistic to expect GPs to maintain sufficient product-level knowledge to optimize individual prescribing, which can perpetuate inappropriate or redundant prescriptions. Unlike licensed medicines, stoma products face no legislative restrictions on direct-to-patient marketing, and promotional activity or free samples may conceivably prompt patients to request additions without specialist oversight.

Transferring prescribing responsibility from GPs to specialist stoma care nurses (SCNs) has been proposed to improve prescribing quality and reduce inefficiency [23,47]. In principle, this is a logical reallocation but, in practice, significant barriers remain. The UK SCN workforce is limited, with estimates suggesting that approximately 600 specialist stoma nurses work across acute, primary, and community care [54]. Based on the upper estimate of 205,000 ostomates, this equates to a nurse-to-patient ratio of approximately 1:342. It could be envisaged that incorporating routine prescription review into SCN duties would therefore substantially increase workload [51]. Recent studies also indicate that ostomate-SCN contact is limited: a Northampton Integrated Care Board survey found 62% of respondents had no SCN contact in over two years [55], consistent with a national survey by Bowles et al. (2022) identifying substantial regional variation in SCN provision across England [56]. Meaningful SCN engagement in prescribing oversight will therefore require significant investment in workforce capacity and service redesign.

There is considerable scope for improved data analytics and algorithmic monitoring of routine prescribing. The NSQIG report surveyed eight Scottish DACs; of the seven that responded, most reported being able to identify ostomates over- or under-ordering specific products, showing that granular, patient-level prescribing data are already captured within the supply chain yet remain underutilized by NHS commissioners [23]. Integrating this into accessible clinical dashboards would enhance prescribing oversight, enable early identification of patients with changing clinical needs (sustained demand increases could serve as a sentinel indicator of appliance failure or skin deterioration warranting SCN assessment), and support proactive identification of patients reluctant to initiate contact themselves—a meaningful shift towards data-driven, anticipatory care. Given that the stoma industry currently appears to hold more granular prescribing oversight than NHS boards, greater formalized collaboration between DACs, commissioners, and SCNs may offer a practical route to improving both prescribing efficiency and patient outcomes.

### 4.2. Limitations of the PCA Data

The major limitation precluding more detailed analysis is the absence of reliable national statistics on the relative proportions of ostomates with an ileostomy, colostomy, or urostomy, constraining analysis of pouch utilization by stoma type and hampering evaluation of geographical prescribing variation. Were this to be addressed, it could help identify both commendable and questionable practices. NSQIG has acknowledged the principal challenges in collating stoma statistics and emphasized the need for more coherent recording of stoma surgeries [23]. The stoma population is dynamic, with a floating cohort who have received a temporary stoma but for whom no robust mechanism exists to record formation or reversal, so interpreting prescribing trends accurately requires a clearer understanding of actual ostomate numbers.

The PCA data also excludes the full cost of maintaining specific ostomate groups in the community. Supplies from district nurses are captured separately, as are specialty medicines—an omission potentially significant for ostomates receiving immunosuppressant biologics such as anti-TNF agents. Home parenteral nutrition (HPN) is a critical therapy for individuals with intestinal failure, and a significant proportion of recipients are likely to also have a stoma [57,58]. Approximately 50 HPN patients per million population are estimated in the UK (close to 3000 individuals [59] with prevalence increasing at roughly 20% per annum and around 30% of recipients on long-term therapy for over five years [60]. Though delivered in the community, HPN costs of approximately £300 per day per patient for compound nutrition alone (excluding flush solutions, dressings, and aseptic ancillaries) are not captured in the PCA dataset.

From a health economics perspective, several evidence gaps are apparent. The absence of reliable data on the relative proportions of colostomy, ileostomy, and urostomy patients significantly constrains interpretation of prescribing data and limits identification of geographical variations in care. A clearer delineation between new and established ostomates would also be valuable, given that prescription requirements differ substantially across the stoma lifecycle; greater early-stage support may reduce longer-term expenditure on complication-related products. It could be envisaged that linking the PCA datasets with hospital episode statistics (HES) or primary care electronic health records (such as CPRD in the UK) could allow researchers to track clinical outcomes (i.e., treatment for peristomal skin complications) alongside prescribing patterns. Prescriber datasets are already available within the system, though not necessarily what has been dispensed. Confirmation of the latter requires greater integration with the NHSBSA PCA database. The CPRD dataset could also provide a more robust measure of the ostomate population. However, the data can be incomplete or ambiguous, as they depend on the information entered and unfortunately incomplete data remains a problem despite good practice guidelines [61].

While the cost–benefit advantage of 2-piece over 1-piece pouch systems is demonstrable in principle for colostomy patients, no studies have examined its real-world, long-term health economics, including impact on PSC rates and healthcare utilization. Some products will also inevitably be used off-label, both in stoma care and in wider wound-care contexts. As noted above, individuals with high-output ileostomies may use urostomy pouch systems; however, pouches can also function as drainage receptacles for wounds and fistulas or as repurposed suction pouches. Similarly, barrier products designed for peristomal skin may be used as bedside workarounds for wounds or to improve seals in negative-pressure therapy devices. Although such adaptations may account for only a small proportion of overall use, particularly because most expenditure relates to pouches, little is known about their prevalence.

## 5. Conclusions

This analysis of UK PCA data for 2023 provides a population-level overview of prescribing patterns in ostomy care and identifies several areas of clinical and health economic significance. The PCA dataset, despite its limitations, represents an underutilized resource for health services research in this field. Continued, more systematic analysis of these datasets, ideally complemented by robust national stoma registry data, has the potential to inform evidence-based prescribing policy, improve resource allocation efficiency, and ultimately support better outcomes for the growing population of individuals living with a stoma in the United Kingdom.

## Figures and Tables

**Table 1:** Prevalence of intestinal or urinary stomas.

Country	Published Estimate	Prevalence %	Ref.
UK (2019)	176,824	0.26	[1]
UK (2022)	205,000	0.31	[2,3,4]
Sweden	43,000	0.41–0.44	[16]
Portugal	19,793	0.19	[17]
United States	700,000–1,000,000	0.21–0.29	[18,19]

**Table 2:** Prescription Cost Analysis (PCA) summary data for the UK National Health Service in 2023.

		England	Northern	Scotland	Wales	Total
	Units		Ireland			UK
Total Annual PCA *	£/million	10,965.5	504.1	1235.6	698.8	13,404.0
Ostomy PCA	£/million	405.4	13.2	28.3	10.1	457.1
Relative Spend	%	3.7	2.6	2.3	1.5	3.4
Population **	Million	57.7	1.9	5.5	3.2	68.3
Ostomates (StoMap)		148,886	4951	14,500	8488	176,824
Ostomates (1/335)		172,210	5733	16,388	9445	203,776
Average Cost per	£	2354–2723	2311–2676	1726–1951	1073–1194	2243–2585
Ostomate						

* Collated from PCA published by each of the devolved nations based on calendar year data; ** Derived from 2023 UK Census Data; Estimate based on 1 ostomate for every 335 people.

**Table 3:** Northern Ireland Stoma Care Accessory Formulary guidelines [25].

Not for Routine Prescription	Prescribed Only After Consultation with a SCN
Deodorisers	Lubricating deodorants
Skin cleansers	Ring seals
Bag/pouch covers	Stoma collars
Barrier creams	Stoma pastes
Stoma filters	Stoma powders
Gauze swabs	
Light support wear	Stoma support wear for parastomal hernia prevention

**Table 4:** UK prescribing guidelines for stoma management in adult patients. (Adapted from: Prescribing Guideline for Management of Stoma (Colostomy, Ileostomy and Urostomy) in adult patients, North East and North Cumbria [26]).

Stoma Type	Type of Appliance	Component	Change Frequency	Quantity
Colostomy	1-Piece Closed	Integrated	1–3× daily	30–90
	2-Piece Closed	Flange	2–3 days	10–15
		Pouch	1–3× daily	30–90
Ileostomy	1-Piece Drainable	Integrated	1–3 days	15–30
	2-Piece Drainable	Flange	2–3 days	10–15
		Pouch	1–3 days	15–30
Urostomy	1-Piece Drainable	Integrated	1–3 days	15–30
	2-Piece Drainable	Flange	2–3 days	10–15
		Pouch	1–3 days	15–30
	Overnight Drainable		Every 7 days	4

## Data Availability

The data used in the compilation of the tables and figures are freely available from the Prescription Cost Analysis pages within the National Health Service Business Services Authorities for each of the devolved nations.
